# Supplementary material for: Adjuvant BRAF-MEK Inhibitors versus Anti PD-1 Therapy in Stage III Melanoma: A Propensity-Matched Outcome Analysis
Source: Cancers (Basel). 2023 Jan 7;15(2):409. doi: 10.3390/cancers15020409 (PMC9857200; doi:10.3390/cancers15020409)
Supplement: Supplementary file 1 [file cancers-15-00409-s001.zip › cancers-2039148-supplementary.pdf]

Supplement

Table S1. Other patient and tumor characteristics in cutaneous melanoma patients treated with adjuvant BRAF/MEK-inhibition therapy and adjuvant anti-PD-1-treated patients.

	Original sample			Nearest neighbor caliper matching		
	**BRAF/MEK-inhibition therapy**	**Anti-PD-1 therapy**	**p-value**	**BRAF/MEK-inhibition therapy**	**Anti-PD-1 therapy**	**p-value**
**N**	114	532	<0.01	112	112	1.00
**Melanoma type (%)**						
Superficial spreading	77 (68.1)	342 (64.3)	0.60	76 (67.9)	68 (60.7)	0.40
Nodular	25 (22.1)	111 (20.9)		24 (21.4)	27 (24.1)	
Acral lentiginous	0 (0.0)	6 (1.1)		0 (0.0)	2 (1.8)	
Other	0 (0.0)	4 (0.8)		0 (0.0)	0 (0.0)	
Unknown	12 (9.7)	69 (13.0)		12 (10.7)	15 (13.4)	
**Melanoma location (%)**			0.84			0.50
Head/Neck	12 (10.5)	66 (12.4)		11 (9.8)	11 (9.8)	
Trunk	61 (53.5)	284 (53.4)		61 (54.5)	69 (61.6)	
Extremity/Acral	41 (36.0)	182 (34.2)		40 (35.7)	32 (28.6)	
**Breslow thickness (in mm) (median [range])**	2.3 [0.3, 27.0]	2.5 [0.1, 32.0]	0.43	2.3 [0.3, 16.3]	2.5 [0.4, 32.0]	0.95
**Ulceration (%)**			0.46			0.16
No	61 (53.5)	312 (58.6)		60 (53.6)	73 (65.2)	
Yes	39 (34.2)	172 (32.3)		14 (12.5)	8 (7.1)	
Unknown	14 (12.3)	48 (9.0)		38 (33.9)	31 (27.7)	
**BRAF mutation (%)**			0.48			0.51
V600E	89 (78.1)	386 (72.6)		87 (77.7)	80 (71.4)	
V600K	11 (9.6)	50 (9.4)		11 (9.8)	13 (11.6)	
V600R	1 (0.9)	13 (2.4)		1 (0.9)	1 (0.9)	
Other BRAF mutation	8 (7.0)	54 (10.2)		8 (7.1)	13 (11.6)	
Unknown BRAF mutation	5 (4.4)	29 (5.5)		5 (4.5)	5 (4.5)	
**In-transit melanoma (%)**			0.12			0.94
No satellite/ITM	81 (71.1)	385 (72.4)		80 (71.4)	79 (70.5)	
ITM only	11 (9.6)	76 (14.3)		10 (8.9)	11 (9.8)	
ITM with nodal involvement	20 (17.5)	69 (13.0)		20 (17.9)	21 (18.8)	
Unknown	2 (1.8)	2 (0.4)		2 (1.8)	1 (0.9)	
**LDH (%)**			<0.01			0.09
Normal	96 (84.2)	504 (94.7)		95 (84.8)	105 (93.8)	
250-500	13 (11.4)	15 (2.8)		13 (11.6)	5 (4.5)	
Unknown	5 (4.4)	13 (2.4)		4 (3.6)	2 (1.8)	

ITM = In-transit melanoma, LDH = lactate dehydrogenase.

Table S2. Comorbidities and comedication in cutaneous melanoma patients treated with adjuvant BRAF/MEK-inhibition therapy and adjuvant anti-PD-1-treated patients.

	**Adjuvant BRAF/MEK- treated patients**	**Adjuvant anti-PD-1**	**p-value**
**n**	114	532	
**Autoimmune Disease (%)**	23 (20.2)	18 (3.4)	**<0.01**
Rheumatoid comorbidities /SLE/scleroderma (%)	12 (10.5)	9 (1.7)	**<0.01**
Morbus Graves (%)	1 (0.9)	0 (0.0)	0.40
IBD (%)	6 (5.3)	1 (0.2)	**<0.01**
Other autoimmune comorbidities (%)	3 (2.6)	4 (0.8)	0.21
**Cardiovascular (%)**	19 (16.7)	65 (12.2)	0.26
**Vascular (%)**	27 (23.7)	119 (22.4)	0.86
**Diabetes Mellitus (%)**	11 (9.6)	47 (8.8)	0.92
**Pulmonary (%)**	10 (8.8)	41 (7.7)	0.84

**Neurological/Psychiatric (%)**	14 (12.3)	63 (11.8)	1.00
**Digestive tract (%)**	13 (11.4)	32 (6.0)	0.07
**Urogenital (%)**	15 (13.2)	44 (8.3)	0.14
**Thromboembolic (%)**	5 (4.4)	7 (1.3)	0.07
**Musculoskeletal (%)**	22 (19.3)	46 (8.6)	**<0.01**
**Endocrine (%)**	6 (5.3)	34 (6.4)	0.81
**Infectious disease (%)**	5 (4.4)	7 (1.3)	0.07
**Previous malignancy (%)**	13 (11.4)	71 (13.3)	0.69
**Transplant (%)**	3 (2.6)	0 (0.0)	**<0.01**
**Other comorbidities (%)**	33 (28.9)	113 (21.2)	0.09
**Comedication (%)**	76 (66.7)	288 (54.1)	**0.02**

SLE= Systemic Lupus Erythematosus, IBD: Inflammable Bowel Disease.

Table S3. Patient characteristics of unmatched patients after nearest neighbor matching.

		**Adjuvant BRAF/MEK-treated patients**	**Adjuvant anti-PD-1**	**p-value**	**SMD**
		2	420		
**Age**	<65	0 (0.0)	270 (64.3)	0.250	1.897
	>65	2 (100.0)	150 (35.7)		
**Sex**	1	0 (0.0)	250 (59.5)	0.323	1.715
	2	2 (100.0)	170 (40.5)		
**ECOG PS**	0	2 (100.0)	318 (75.7)	1.000	0.801
	>=1	0 (0.0)	102 (24.3)		
**Comorbidities**	No	2 (100.0)	168 (40.0)	0.316	1.732
	Yes	0 (0.0)	252 (60.0)		
**AJCC8 Stage**	IIIA	0 (0.0)	36 (8.6)	0.440	1.649
	IIIB	0 (0.0)	169 (40.2)		
	IIIC/D	2 (100.0)	178 (42.4)		
	Unknown	0 (0.0)	37 (8.8)		

Table S4. Patient characteristics after optimal matching.

		**BRAF/MEK-inhibition therapy**	**Anti-PD-1 therapy**	**p-value**	**SMD**
		114	114		
**Age**	<65	76 (66.7)	71 (62.3)	0.580	0.092
	>65	38 (33.3)	43 (37.7)		
**Sex**	1	63 (55.3)	65 (57.0)	0.894	0.035
	2	51 (44.7)	49 (43.0)		
**ECOG PS**	0	82 (71.9)	82 (71.9)	1.000	<0.001
	>=1	32 (28.1)	32 (28.1)		
**Comorbidities**	No	29 (25.4)	27 (23.7)	0.878	0.041
	Yes	85 (74.6)	87 (76.3)		
**AJCC8 Stage**	IIIA	18 (15.8)	19 (16.7)	0.998	0.025
	IIIB	31 (27.2)	31 (27.2)		
	IIIC/D	51 (44.7)	50 (43.9)		
	Unknown	14 (12.3)	14 (12.3)		

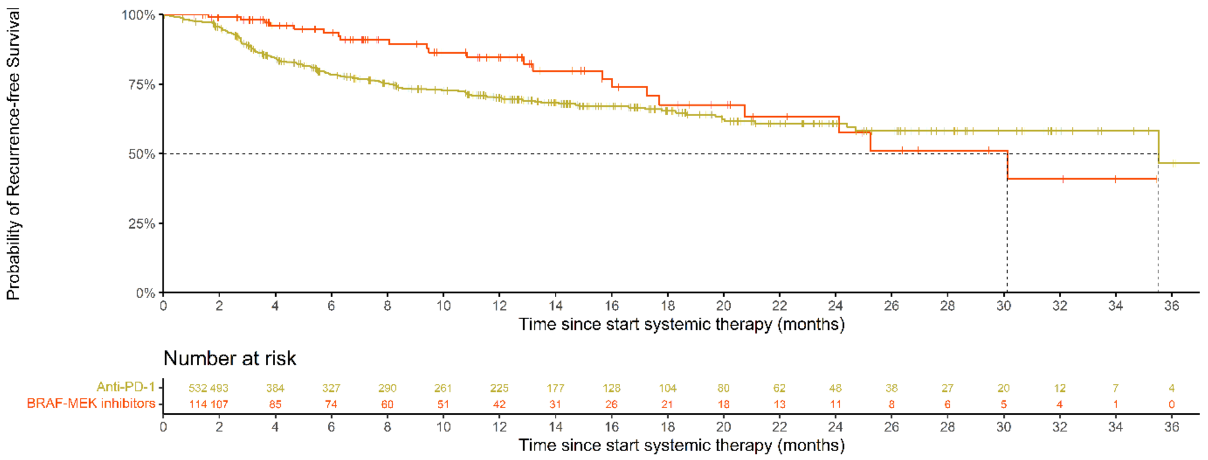

Figure S1: Recurrence-free survival (RFS) of BRAF/MEK- and anti-PD-1-treated patients before propensity score matching in the original cohort.

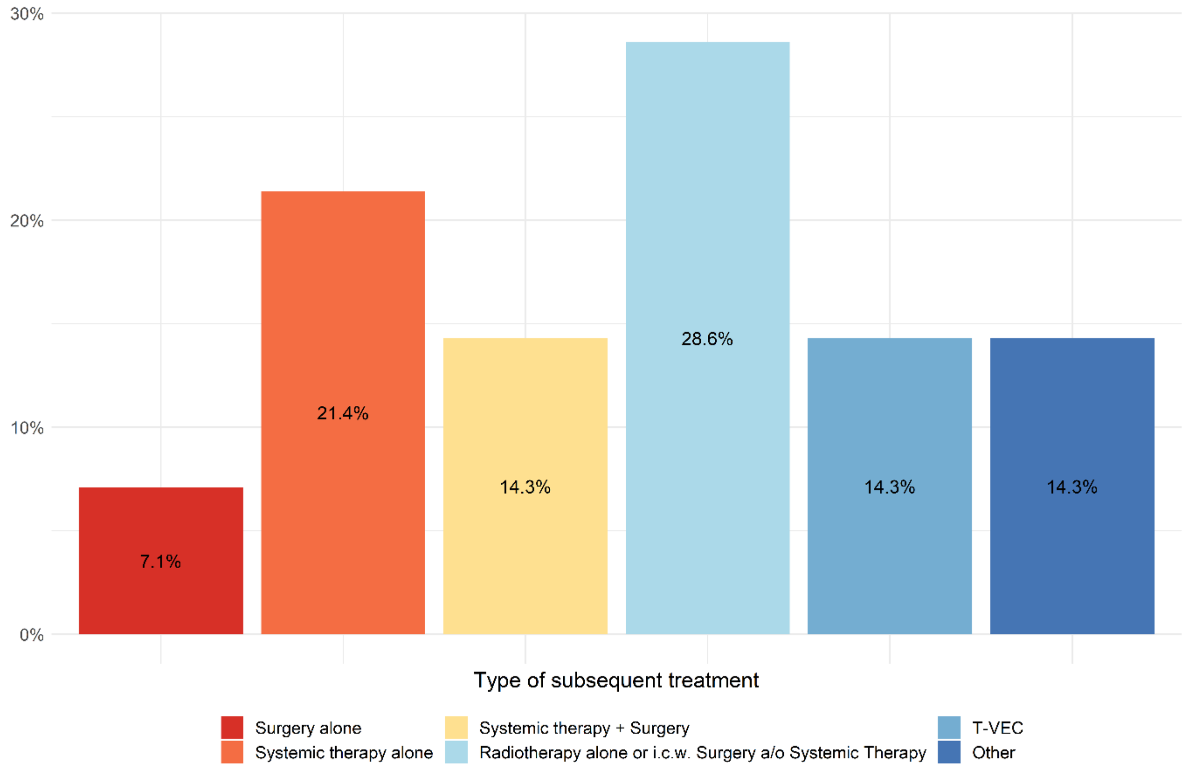

Figure S2: Type of subsequent treatment in patients in BRAF/MEK-treated patients who had a subsequent treatment registered into the DMTR database. (n=14)

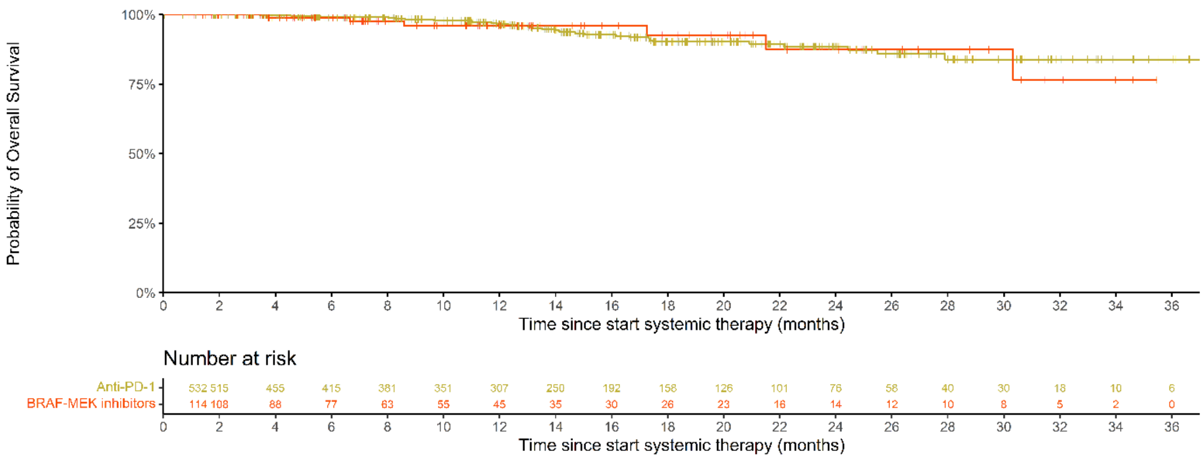

Figure S3: Overall survival (OS) of BRAF/MEK- and anti-PD-1-treated patients before propensity score matching in the original cohort.

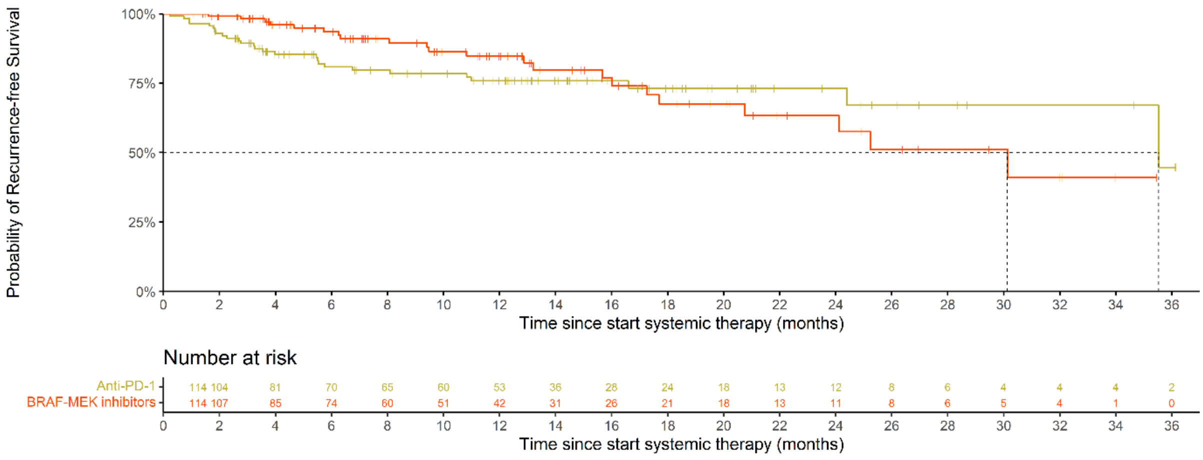

Figure S4: Recurrence-free survival (RFS) of BRAF/MEK- and anti-PD-1-treated patients after optimal matching.

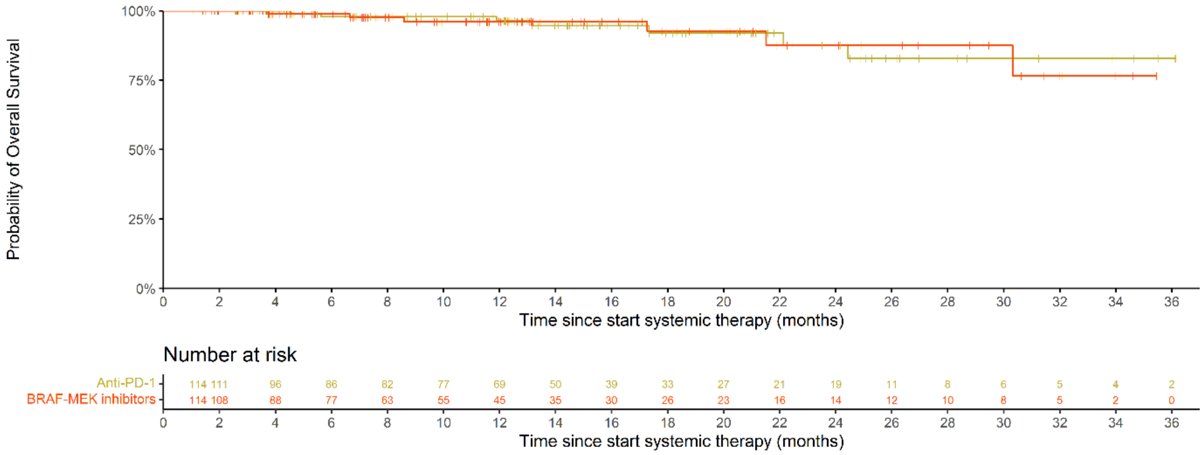

Figure S5: Overall survival (OS) of BRAF/MEK- and anti-PD-1-treated patients after optimal matching.
